# Impact of patient positioning uncertainty in noncoplanar intracranial stereotactic radiotherapy

**DOI:** 10.1002/acm2.12820

**Published:** 2020-01-20

**Authors:** Yoshihiro Tanaka, Masataka Oita, Shinichiro Inomata, Toshiaki Fuse, Yuichi Akino, Kohei Shimomura

**Affiliations:** ^1^ Department of Radiation Therapy Japanese Red Cross Society Kyoto Daiichi Hospital Kyoto Japan; ^2^ Department of Healthcare Sciences Graduate School of Interdisciplinary Science and Engineering in Health Systems Okayama University Okayama Japan; ^3^ Oncology Center Osaka University Hospital Osaka Japan; ^4^ Kyoto College of Medical Science Kyoto Japan

**Keywords:** IGRT, noncoplanar radiotherapy, patient positioning uncertainty, SRS, SRT

## Abstract

The aim of this study is to evaluate the patient positioning uncertainty in noncoplanar stereotactic radiosurgery or stereotactic radiotherapy (SRS/SRT) for intracranial lesions with the frameless 6D ExacTrac system. In all, 28 patients treated with SRS/SRT of 70 treatment plans at our institution were evaluated in this study. Two X‐ray images with the frameless 6D ExacTrac system were first acquired to correct (XC) and verify (XV) the patient position at a couch angle of 0º. Subsequently, the XC and XV images were also acquired at each planned couch angle for using noncoplanar beams to detect position errors caused by rotating a couch. The translational XC and XV shift values at each couch angle were calculated for each plan. The percentages of the translational XC shift values within 1.0 mm for each planned couch angle for using noncoplanar beams were 77.86%, 72.26%, and 98.47% for the lateral, longitudinal, and vertical directions, respectively. Those within 2.0 mm were 98.22%, 97.96%, and 99.75% for the lateral, longitudinal, and vertical directions, respectively. The maximum absolute values of the translational XC shifts among all planned couch angles for using noncoplanar beams were 2.69, 2.45, and 2.17 mm for the lateral, longitudinal, and vertical directions, respectively. The overall absolute values of the translational XV shifts were less than 1.0 mm for all directions except for one case in the longitudinal direction. The patient position errors were detected after couch rotation for using noncoplanar beams, and they exceeded a planning target volume (PTV) margin of 1.0–2.0 mm used commonly in SRS/SRT treatment. These errors need to be corrected at each planned couch angle, or the PTV margin should be enlarged.

## 1 INTRODUCTION

Stereotactic radiosurgery or stereotactic radiotherapy (SRS/SRT) for intracranial benign and malignant lesions has been a well‐established technique and a standard modality for many years.^1^ SRS/SRT prescribes a high dose for one to five fractions, and a planning target volume (PTV) margin for a clinical target volume (CTV) is typically set as 1.0–2.0 mm.^2^ The accuracy of the treatment planning and delivery is essential in dealing with local tumor and spare normal tissues. Modern advanced radiotherapy techniques such as dynamic conformal arc (DCA), intensity‐modulated radiotherapy (IMRT), and volumetric‐modulated arc therapy (VMAT) have enabled steep dose falloff around target volumes while sparing organ at risk (OAR),^3^, ^4^ whereas we have an increased need for the necessity of more accurate patient setup and treatment delivery. Treuer et al. concluded that the upper limit as a safety margin of target point deviations was 1.3 mm in SRS for arteriovenous malformation (AVM) and brain metastases.^5^

Conventionally, using invasive fixation devices to the patient's skull, such as metal frames or rings were essential for patient immobilization and target localization in SRS/SRT treatment of intracranial lesions.^6^ However, noninvasive (frameless) SRS/SRT treatment has become a standard procedure owing to the development of image‐guided radiotherapy (IGRT) systems in recent years.^7^, ^8^, ^9^, ^10^ Chang et al. reported that the accuracy of the patient setup with cone‐beam computed tomography (CBCT) image guidance was comparable to that with frame‐based radiosurgery systems.^7^ The frameless 6D ExacTrac system (BrainLAB A.G., Heimstetten, Germany), which is mainly an integration of an infrared (IR)‐based optical positioning system and a radiographic kV X‐ray imaging system, is one of the advanced IGRT system.^11^, ^12^, ^13^ Keeling et al. evaluated the patient setup accuracy of SRS/SRT with this system of 35 patients with cranial lesions using the positioning shift values in this system. They reported that the residual setup errors at a couch angle of 0º after positioning correction were less than 0.3 mm and 0.3º in the translational and rotational directions, respectively.^13^ Furthermore, the 6D ExacTrac system can be used even when a couch is rotated using noncoplanar beams. They also investigated the couch sagging shifts for various couch angles and weights at a phantom study and defined the quadrature sum of the translational couch position uncertainties as to the couch sagging uncertainty. Moreover, they reported that the quadrature sum of the translational uncertainties (maximum value was 1.09 mm at couch angle of 270° using a weight of 70 kg) mostly depended on couch angles rather than weights of the phantom. Noncoplanar beams (or arcs) are commonly used in SRS/SRT treatment with a linear accelerator to improve dose conformity and normal tissue sparing.^14^, ^15^ Murphy et al. analyzed the patterns of patient movements during frameless image‐guided radiosurgery with the CyberKnife. They showed that the translational patient position shifts added up to 2 mm throughout a cranial treatment site.^16^ However, the radiation isocenter itself might be shifted at random by the contributions of noncoplanar beams and the weight of a patient whenever a couch is rotated. Although some reports showed the high detection of patient position errors and positioning accuracy using the frameless 6D ExacTrac system,^17^, ^18^ no study were precisely dealing with the uncertainties in case of rotating a couch with noncoplanar beams.

In this study, we first evaluated the accuracy of the patient setup in SRS/SRT treatment for intracranial lesions using the frameless 6D ExacTrac system at our institution. Then, the translational patient position errors caused by the rotation of a couch to the planned position using noncoplanar beams, which were analyzed from a database recorded for each patient’s plan.

## 2 MATERIALS AND METHODS

### 2.1 ExacTrac system and procedure of the patient setup

**Figure 1:**
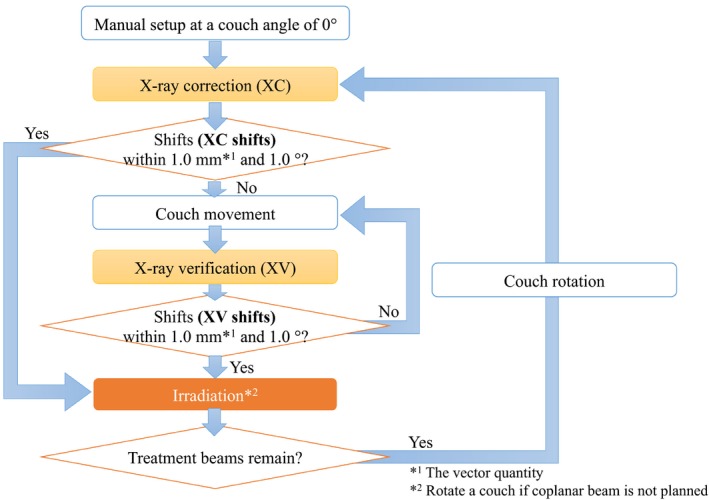
The flowchart of patient setup procedure in stereotactic radiosurgery or stereotactic radiotherapy for intracranial lesions with the frameless 6D ExacTrac system.

In this study, a Novalis‐Tx™ linear accelerator (Varian Medical Systems and BrainLAB A.G., Heimstetten, Germany) with the ExacTrac system version 6.0.6 (BrainLAB A.G., Heimstetten, Germany) was used. Figure 1 shows the flowchart of the patient setup procedure. First, at a couch angle of 0º, we set up the patient manually at the isocenter position. Next, two X‐ray images (X‐ray correction: XC) were acquired and matched with reference digitally reconstructed radiographs (DRRs) created by the ExacTrac software using the computed tomography (CT) simulation images. CT scan parameters were set as follows: The X‐ray tube voltage, slice thickness, and field‐of‐view values were 120 kV, 1.0 mm, and 500 mm, respectively, and the mAs value was determined by an auto‐exposure control function. This matching system was applied the rigid image fusion with the bone anatomy and calculated the necessary translational and rotational 6D couch shift values for moving the patient to the isocenter position. If the calculated position error values (XC shifts) exceeded our institutional criteria, which are within 1.0 mm in a vector quantity and 1.0º for the translational and rotational shift values, respectively, the couch position was corrected using the IR guidance system by monitoring IR reflective markers attached to the cranial positioning array (BrainLAB A.G., Heimstetten, Germany). Subsequently, the second set of two X‐ray images (X‐ray verification: XV) was acquired to validate that the moved patient translational and rotational positions were within our institutional criteria. However, the XV was not acquired if the XC shifted below our institutional criteria. This process was repeated until all position error values (XV shifts) were within our institutional criteria.

### 2.2 Geometric accuracy of the linear accelerator, the ExacTrac system, and the treatment couch

**Figure 2:**
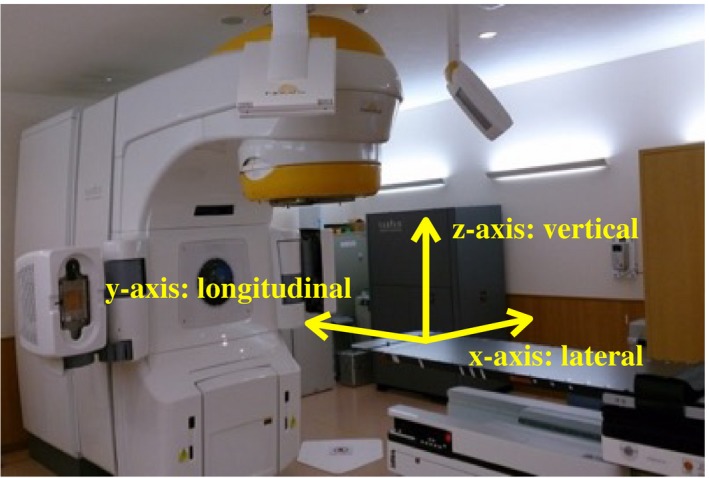
The schema for the couch or patient position coordinate system in the ExacTrac system where x‐, y‐, and z‐axes are lateral, longitudinal, and vertical shifts, respectively.

According to the recommendations of the American Association of Physicists in Medicine (AAPM) Task Group 142,^19^ quality assurance (QA) evaluations of the linear accelerator and ExacTrac system were carried out daily, monthly, and annually. The isocenter position of the IR and X‐ray imaging acquired from the ExacTrac system was tested with an isocenter calibration phantom and isocenter pointer phantom provided by the vendor, respectively. An isocentric coincidence at our institution, including lasers, light, radiation, IR, and X‐rays, was within the SRS tolerance of 1.0 mm (data not shown). Moreover, Winston‐Lutz (WL) test^20^ was performed by the vendor as to the evaluation by the independent organization in April 2017 and May 2018. Then, the mean and standard deviation (SD) values of the position offsets for the gantry, collimator, and couch rotations were less than 0.5 mm, as shown in Table 1. Figure 2 shows the schematic of the couch coordinate system for the ExacTrac system, differing from the linear accelerator coordinate system, and this coordinate system rotates with a couch. The ExacTrac software calculates the XC and XV shifts in this coordinate system attached to each couch position.

**Table 1:** Mean and standard deviation (SD) values of offset for the gantry, the collimator, and the couch rotations derived by vendor’s Winston‐Lutz test.

Radiation Isocenter Measurements (Mean ± 1 SD) [mm]						
	Year 2017			Year 2018		
	Lat.	Long.	Vert.	Lat.	Long.	Vert.
Gantry Radiation Isocenter	0.05 ± 0.11	0.02 ± 0.26	−0.01 ± 0.11	−0.01 ± 0.17	−0.01 ± 0.25	−0.01 ± 0.16
Collimator Radiation Isocenter	0.02 ± 0.03	−0.27 ± 0.04	n/a	−0.10 ± 0.03	−0.32 ± 0.00	n/a
Couch Radiation Isocenter	0.03 ± 0.32	−0.09 ± 0.25	n/a	0.01 ± 0.11	−0.28 ± 0.13	n/a

Abbreviations: Lat., lateral; Long., longitudinal; Vert., vertical.

**Figure 3:**
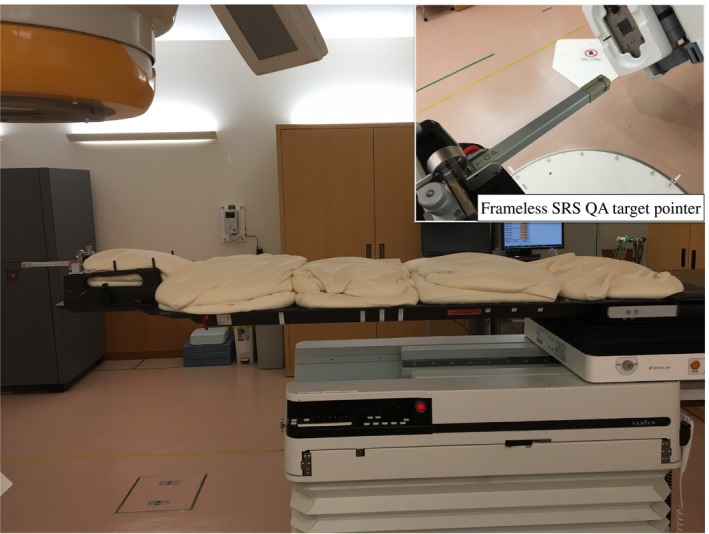
The aspect of the frameless SRS QA target pointer on the weighted couch with a phantom study.

Based on the previous report^13^, we evaluated the translational couch position uncertainties in our institution for some couch angles and weights with a phantom study. As shown in Fig. 3, we set weights (0, 40, 60, and 80 kg) on a treatment couch evenly and carried out a WL tests for couch rotation angles (0, 45, 90, 270, and 315°) using weighted couch and a frameless SRS QA target pointer (BrainLAB A.G., Heimstetten, Germany). For each couch rotation angle, the offset value of the WL sphere (XC shifts in Fig. 1) was calculated using the ExacTrac software, and the value subtracted by that of the reference couch angle of 0º was defined as the translational couch position uncertainties. Table 2 summarized the position offset values for couch angles and weights. All offset values were within the SRS tolerance of 1.0 mm, even when a couch was weighted.

**Table 2:** Offset values for the couch rotation at typical couch angles and weights in the phantom study.

Weight [kg]	Couch Angle [º]	Lat. [mm]	Long. [mm]	Vert. [mm]
0	90	−0.75	0.25	−0.03
	45	−0.32	−0.14	−0.02
	270	0.40	0.77	−0.01
	315	0.44	0.45	−0.02
40	90	−0.81	0.19	−0.07
	45	−0.34	−0.20	−0.03
	270	0.26	0.80	−0.06
	315	0.43	0.47	−0.06
60	90	−0.85	0.33	−0.06
	45	−0.39	−0.12	−0.04
	270	0.38	0.90	−0.05
	315	0.52	0.54	−0.06
80	90	−0.72	0.43	−0.05
	45	−0.34	−0.05	−0.06
	270	0.45	0.78	−0.06
	315	0.53	0.46	−0.06

Abbreviations: Lat., lateral; Long., longitudinal; Vert., vertical.

### 2.3 Patient immobilization

A noninvasive thermoplastic mask (BrainLAB A.G., Heimstetten, Germany) was used to immobilize all patients in SRS/SRT for intracranial lesions. A previous report mentioned descriptions of this thermoplastic mask system.^13^ In this study, we did not use a localizer box in the CT simulation, but we marked the CT origin coordinate on the noninvasive thermoplastic mask directly as same as the conventional radiation therapy procedure. However, as mentioned in the section of the procedure of patient setup and the flowchart, we considered that the uncertainties of CT origin coordinate caused by this procedure could not be affected at the manual setup at the couch angle of 0° for the evaluation of the initial reference position.

### 2.4 Data analysis

Between January 2017 and August 2018, 28 patients having intracranial lesions treated with SRS/SRT at our institution were included in this study. A total of 70 treatment plans were implemented because some plans had multiple isocenters. It was possible to use the ExacTrac system even when a couch position was located at noncoplanar angles. Therefore, we acquired the XC and XV at each planned couch angle to confirm the patient’s position whenever we rotated the couch for using noncoplanar beams. However, the XV was not acquired if the XC shifts were within our institution’s criteria, as shown in Fig. 1. The translational XC and XV shifts were collected in the CT database for each patient plan. The XC and XV shifts were defined as the calculated shift values based on the first and last two X‐ray images for each couch angle to irradiate in this study.

First, to evaluate the accuracy of the patient setup at our institution, we calculated the mean and SD values of the translational XC and XV shifts at a couch angle of 0º for each patient plan. If a patient had treated only one fraction for the SRS treatment, single translational XC and XV shifts were used as the mean values, and SD values of zero mm. In addition, the systematic and random errors proposed by Bijhold et al. were calculated for the entire group.^21^ Then, the translational XC and XV shifts at each planned couch angle for using noncoplanar beams were evaluated to investigate the impact of the patient position errors by the couch rotation. Here, to compare them in the couch coordinate axes at a couch angle of 0º, that is, the Cartesian coordinate system, we converted those for the lateral and longitudinal directions to the Cartesian coordinate system. We defined the XC or XV shifts at each planned couch angle of the noncoplanar beam for the lateral and longitudinal directions in the Cartesian coordinate system as $Lat\left(x\right)$ and $Lng\left(y\right)$, which were calculated as follows:

(1) $$Lat\left(x\right)=\sqrt{x^{2}+y^{2}}\;\sin\left\{\tan^{-1}\left(\frac{y}{x}\right)+2\pi -\theta \right\},$$

(2) $$Lng\left(y\right)=\sqrt{x^{2}+y^{2}}\;\cos\left\{\tan^{-1}\left(\frac{y}{x}\right)+2\pi -\theta \right\},$$

where x and y indicated each data of the XC or XV shifts for the lateral and longitudinal directions in each couch coordinate system, and θ [rad] denoted the couch rotation angle. If y was a negative value, the signs of both $Lat\left(x\right)$ and $Lng\left(y\right)$ were reversed.

## 3 RESULTS

**Figure 4:**
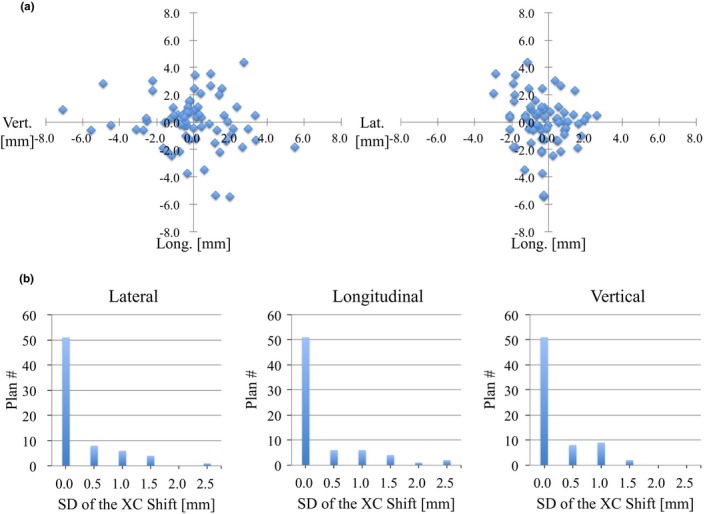
(a) Mean and (b) standard deviation (SD) of translational XC shifts (lateral, longitudinal, and vertical directions) at the couch angle of 0º for all 70 patient plans.

**Figure 5:**
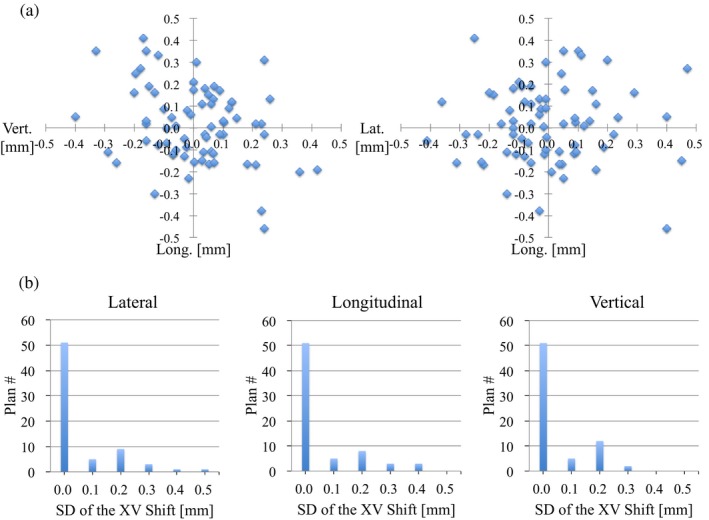
(a) Mean and (b) standard deviation (SD) of translational XV shifts (lateral, longitudinal, and vertical directions) at the couch angle of 0º for all 70 patient plans.

Figures 4 and 5 show the mean and SD values of the translational XC and XV shifts at a couch angle of 0º for each patient plan. Although most of the mean values of the translational XC shifts were from −5.0 to +5.0 mm, all those of the translational XV shifts were from −0.5 to +0.5 mm. The SD values of the translational XC and XV shifts were within 2.5 and 0.4 mm for all cases, respectively. Table 3 summarizes the systematic and random errors calculated from the translational XC and XV shifts at a couch angle of 0º for all 70 patient plans. The systematic and random errors with the translational XC shifts in the entire group were 1.18, 1.85, and 2.08 mm, and 0.46, 0.58, and 0.35 mm for the lateral, longitudinal, and vertical directions, respectively. Those for the translational XV shifts were less than 0.2 and 0.1 mm in all three directions.

**Table 3:** Systematic and random errors calculated from translational XC and XV shifts at the couch angle of 0º for all 70 patient plans.

	XC Shift [mm]			XV Shift [mm]		
	Lat.	Long.	Vert.	Lat.	Long.	Vert.
Systematic errors	1.18	1.85	2.08	0.18	0.18	0.16
Random errors	0.46	0.58	0.35	0.09	0.10	0.08

Abbreviations: XC, X‐ray correction; XV, X‐ray verification; Lat., lateral; Long., longitudinal; Vert., vertical.

**Figure 6:**
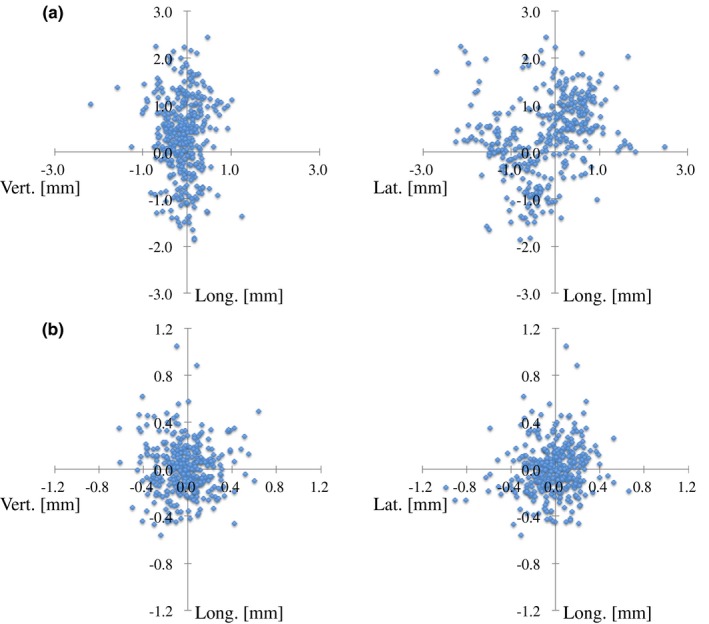
Scatter plots of translational (a) XC and (b) XV shifts in the Cartesian coordinate system for each planned couch angle for use with 362 noncoplanar beams.

**Figure 7:**
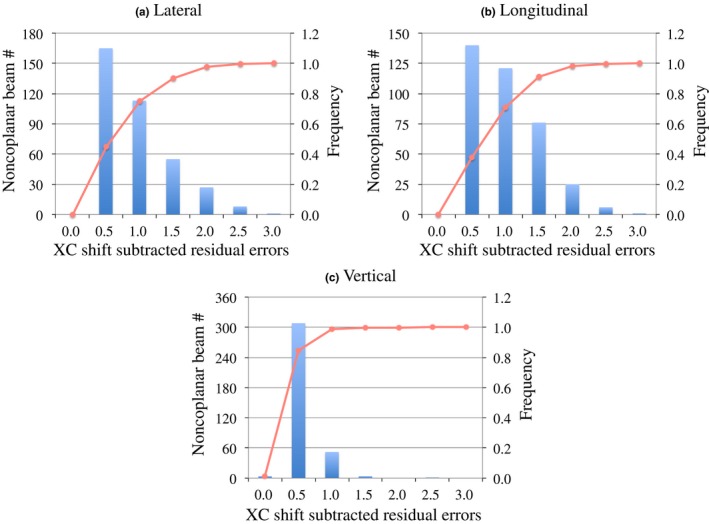
Histograms and frequencies of absolute values of translational XC shifts subtracted the residual position errors in the Cartesian coordinate system for (a) lateral, (b) longitudinal, and (c) vertical directions for all planned couch angles for use with 362 noncoplanar beams.

Figure 6 illustrates the translational XC and XV shifts in the Cartesian coordinate system for all couch angles planned for use with 362 noncoplanar beams, and indicates that many translational XC shifts exceeded the PTV margin of 1.0 or 2.0 mm. Table 4 summarizes the mean, SD, and Max values of the absolute values of the translational XC and XV shifts in the Cartesian coordinate system. For the translational XC shifts, although both the mean and SD values were less than 1.0 mm in all directions, the Max values were 2.69, 2.45, and 2.17 mm in the lateral, longitudinal, and vertical directions, respectively. Figure 7 shows the histograms and frequencies of the absolute values of the translational XC shifts subtracted the residual position errors in the Cartesian coordinate system for all couch angles planned for use with 362 noncoplanar beams. The percentages of the translational XC shifts within 1.0 mm were 75.3%, 70.7%, and 98.6% in the lateral and longitudinal directions, respectively. Those within 2.0 mm were 97.6%, 98.1%, and 99.7% for the lateral, longitudinal, and vertical directions, respectively. All absolute values of the translational XV shifts were less than 1.0 mm in all three directions except for one case in the longitudinal direction.

**Table 4:** Mean, standard deviation (SD), and maximum (Max) values of absolute values of translational XC and XV shifts in the Cartesian coordinate system for all planned couch angles for the use of 362 noncoplanar beams.

	XC Shift [mm]			XV Shift [mm]		
	Lat.	Long.	Vert.	Lat.	Long.	Vert.
Mean	0.69	0.74	0.29	0.16	0.16	0.15
SD	0.52	0.52	0.26	0.14	0.14	0.13
Max	2.69	2.45	2.17	0.99	1.05	0.64

Abbreviations: XC, X‐ray correction; XV, X‐ray verification; Lat., lateral; Long., longitudinal; Vert., vertical.

## 4 DISCUSSION

In this study, we investigated the impacts of the couch rotation to the planned couch angle for using noncoplanar beams on patient position errors in SRS/SRT with the frameless 6D ExacTrac system using a database recorded for each patient plan. Figures 4 and 5 show the accuracy of the patient setup at a couch angle of 0º for SRS/SRT treatment at our institution. Keeling et al. reported that the mean translational XC shifts for each of 49 patient treatments were almost from − 2.5 to + 2.5 mm, and those SD values were much less than 1.0 mm in SRS/SRT with the frameless 6D ExacTrac system.^13^ It was suggested that our results were larger than theirs because a localizer box was not used in the CT simulation, and all patients were set up at the isocenter position manually in the SRS/SRT treatments at our institution. These position errors might not be a major problem, as they could be corrected using the ExacTrac system and software. The mean and SD values of the translational XV shifts were less than ± 0.5 and 0.4 mm, respectively. Infusino et al. reported that the systematic and random errors were measured to be 1.33, 1.73, and 2.30 mm, and 0.20, 0.27, and 0.18 mm in the lateral, longitudinal, and vertical directions, respectively, in SRT for brain tumors with the frameless 6D ExacTrac system.^12^ It is assumed that the accuracy of the patient setup at our institution was equal to theirs (Table 3).

Our analysis results showed that patient position errors over 1.0 or 2.0 mm occurred after rotating a couch to the planned angle for using noncoplanar beams (Fig. 6). In particular, in the lateral and longitudinal directions, the percentages of the XC shifts in the Cartesian coordinate system within 1.0 mm were only 77.9% and 72.3%, respectively. The Max absolute values of the patient position errors were over 2.0 mm in all three directions and deviated from the PTV margin. Similar results also obtained for the translational XC shifts subtracted the residual position errors in the Cartesian coordinate system (Fig. 7), while the translational couch position uncertainties for various couch angles and weights were within the SRS tolerance of 1.0 mm in a phantom study, as shown in Table 2. The previous report illustrated that the patient movement during image‐guided radiosurgery was observed by 2.0 mm for the cranial treatment,^16^ and another showed that translational couch position uncertainties were dependent on the couch angle.^13^ Therefore, the position errors detected in this study might be caused by both the patient’s intra‐fractional motion and the couch angle dependence of the couch position accuracy. Especially, the translational couch position uncertainties for couch angles of 90 and 270° were >0.7 mm, and patient position errors could exceed 1.0 mm by the slightest patient motion. Takakura et al. reported that the accuracy in positional correction with the ExacTrac robotic couch was 0.07 ± 0.22 mm in their phantom study.^22^ Ma et al. compared the residual setup errors with the frameless 6D ExacTrac system and CBCT for a head phantom and 18 patients receiving intracranial SRT, and reported that the root mean square (RMS) of the differences observed for translations was typically <0.5 mm for the phantom and <1.5 mm for the patients.^23^ The patient setup using the frameless 6D ExacTrac system in SRS/SRT for intracranial lesions is extremely accurate, but patient position errors occur when the couch is rotated for using noncoplanar beams. Therefore, implementation of the IGRT requires at each planned couch angle, or a PTV margin is enlarged by more than 2.0 mm in the clinically acceptable tolerance.

The immobilization accuracy affects the patient’s intra‐fractional motion during treatment, but the precision of each noninvasive thermoplastic mask could not be verified in this study. The setup skill and the carefulness of the member of staff also might depend on the accuracies, and the uncertainties of the IR guidance system influence the couch correction shift reported by the past study.^24^ An investigation into the impacts of these factors on patient position errors will be our future work.

## 5 CONCLUSIONS

By rotating a couch for using noncoplanar beams, patient position errors occurred and exceeded a PTV margin in SRS/SRT treatment when using the frameless 6D ExacTrac system. The patient position errors needed to be corrected using IGRT systems at each couch angle. If the IGRT system cannot be used at noncoplanar couch angles, a PTV margin should be enlarged to the clinically acceptable tolerance.

## CONFLICT OF INTEREST

The authors have no conflict of interest to disclose.

## References

[1] Lightstone AW , Benedict SH , Bova FJ , et al. Intracranial stereotactic positioning systems: report of the American Association of Physicists in Medicine Radiation Therapy Committee Task Group No. 68. Med Phys. 2005;32:2380–2398. 10.1118/1.1945347 16121596

[2] Yamazaki H , Shiomi H , Tsubokura T , et al. Quantitative assessment of inter‐observer variability in target volume delineation on stereotactic radiotherapy treatment for pituitary adenoma and meningioma near optic tract. Radiat Oncol. 2011;6:1–6. 21272369 10.1186/1748-717X-6-10 PMC3040152

[3] Ezzell GA , Burmeister JW , Dogan N , et al. IMRT commissioning: multiple institution planning and dosimetry comparisons, a report from AAPM Task Group 119. Med Phys. 2009;36:5359–5373. 19994544 10.1118/1.3238104

[4] Bedford JL , Warrington AP . Commissioning of volumetric modulated arc therapy (VMAT). Int J Radiat Oncol Biol Phys. 2009;73:537–545. 19147018 10.1016/j.ijrobp.2008.08.055

[5] Treuer H , Kocher M , Hoevels M , et al. Impact of target point deviations on control and complication probabilities in stereotactic radiosurgery of AVMs and metastases. Radiother Oncol. 2006;81:25–32. 17005278 10.1016/j.radonc.2006.08.022

[6] Otto K , Fallone FBG . Frame slippage verification in stereotactic radiosurgery. Int J Radiat Oncol Biol Phys. 1998;41:199–205. 9588934 10.1016/s0360-3016(98)00005-4

[7] Chang J , Yenice KM , Narayana A , et al. Accuracy and feasibility of cone beam computed tomography for stereotactic radiosurgery setup. Med Phys. 2007;34:2077–2084. 17654911 10.1118/1.2731031

[8] Ramakrishna N , Rosca F , Friesen S , et al. A clinical comparison of patient setup and intra‐fraction motion using frame‐based radiosurgery versus a frameless image‐guided radiosurgery system for intracranial lesions. Radiother Oncol. 2010;95:109–115. 20116123 10.1016/j.radonc.2009.12.030

[9] Nyflot MJ , Cao N , Meyer J , et al. Improved accuracy for noncoplanar radiotherapy: an EPID‐based method for submillimeter alignment of linear accelerator table rotation with MV isocenter. J Appl Clin Med Phys. 2014;15:151–159. 10.1120/jacmp.v15i2.4682 PMC5875467 24710457

[10] Calvo Ortega JF , Wunderink W , Delgado D , et al. Evaluation of the setup margins for cone beam computed tomography‐guided cranial radiosurgery: a phantom study. Med Dosim. 2016;41:199–204. 26994824 10.1016/j.meddos.2015.12.006

[11] Ali I , Tubbs J , Hibbitts K , et al. Evaluation of the setup accuracy of a stereotactic radiotherapy head immobilization mask system using kV on‐board imaging. J Appl Clin Med Phys. 2010;11:26–37. 10.1120/jacmp.v11i3.3192 PMC5720447 20717086

[12] Infusino E , Trodella L , Ramella S , et al. Estimation of patient setup uncertainty using BrainLAB Exactrac X‐ray 6D system in image‐guided radiotherapy. J Appl Clin Med Phys. 2015;16:99–107. 10.1120/jacmp.v16i2.5102 PMC5690103 26103179

[13] Keeling V , Hossain S , Jin H , et al. Quantitative evaluation of patient setup uncertainty of stereotactic radiotherapy with the frameless 6D ExacTrac system using statistical modeling. J Appl Clin Med Phys. 2016;17:111–127. 10.1120/jacmp.v17i3.5959 PMC5690915 27167267

[14] Meeks SL , Pukala J , Ramakrishna N , et al. Radiosurgery technology development and use. J Radiosurg SBRT. 2011;1:21–29. 29296294 PMC5658896

[15] Yu VY , Tran A , Nguyen D , et al. The development and verification of a highly accurate collision prediction model for automated noncoplanar plan delivery. Med Phys. 2015;42:6457–6467. 26520735 10.1118/1.4932631 PMC4608969

[16] Murphy MJ , Chang SD , Gibbs IC , et al. Patterns of patient movement during frameless image‐guided radiosurgery. Int J Radiat Oncol Biol Phys. 2003;55:1400–1408. 12654453 10.1016/s0360-3016(02)04597-2

[17] Jin JY , Ryu S , Faber K , et al. 2D/3D Image fusion for accurate target localization and evaluation of a mask based stereotactic system in fractionated stereotactic radiotherapy of cranial lesions. Med Phys. 2006;33:4557–4566. 17278807 10.1118/1.2392605

[18] Gevaert T , Verellen D , Tournel K , et al. Setup accuracy of the Novalis ExacTrac 6DOF system for frameless radiosurgery. Int J Radiat Oncol Biol Phys. 2012;82:1627–1635. 21477937 10.1016/j.ijrobp.2011.01.052

[19] Klein EE , Hanley J , Bayouth J , et al. Task group 142 report: Quality assurance of medical accelerators. Med Phys. 2009;36:4197–4212. 19810494 10.1118/1.3190392

[20] Lutz W , Winston KR , Maleki N . A system for stereotactic radiosurgery with a linear accelerator. Int J Radiat Oncol Biol Phys. 1988;14:373–381. 3276655 10.1016/0360-3016(88)90446-4

[21] Bijhold J , Lebesque JV , Hart AA , et al. Maximizing setup accuracy using portal images as applied to a conformal boost technique for prostate cancer. Radiother Oncol. 1992;24:261–271. 1410582 10.1016/0167-8140(92)90233-k

[22] Takakura T , Mizowaki T , Nakata M , et al. The geometric accuracy of frameless stereotactic radiosurgery using a 6D robotic couch system. Phys Med Biol. 2010;55:1–10. 19949261 10.1088/0031-9155/55/1/001

[23] Ma J , Chang Z , Wang Z , et al. ExacTrac X‐ray 6 degree‐of‐freedom image‐guidance for intracranial non‐invasive stereotactic radiotherapy: comparison with kilo‐voltage cone‐beam CT. Radiother Oncol. 2009;93:602–608. 19846229 10.1016/j.radonc.2009.09.009

[24] Wang LT , Solberg TD , Medin PM , et al. Infrared patient positioning for stereotactic radiosurgery of extracranial tumors. Comput Bio Med. 2001;31:101–111. 11165218 10.1016/s0010-4825(00)00026-3

